# Supplementary material for: Cyclic [Cu-biRadical]2 Secondary Building Unit in 2p-3d and 2p-3d-4f Complexes: Crystal Structure and Magnetic Properties
Source: Molecules. 2023 Mar 9;28(6):2514. doi: 10.3390/molecules28062514 (PMC10058193; doi:10.3390/molecules28062514)
Supplement: Supplementary file 1 [file molecules-28-02514-s001.zip › molecules-2248367-supplementary.pdf]

Supplementary Material

Cyclic [Cu-biRadical]$_2$ Secondary Building Unit in 2p-3d and 2p-3d-4f Complexes: Crystal Structure and Magnetic Properties

Xiao-Tong Wang,[a] Xiao-Hui Huang,[a] Hong-Wei Song,[a] Yue Ma,[a] Li-Cun Li*[a] and Jean-Pascal Sutter*[b]

[1] *Department of Chemistry, Key Laboratory of Advanced Energy Materials Chemistry, College of Chemistry, Nankai University, Tianjin 300071, China*

[2] *Laboratoire de Chimie de Coordination du CNRS (LCC-CNRS), Université de Toulouse, Centre national de la recherche scientifique (CNRS), 31077, Toulouse, France*

Table S1. Selected bond lengths [Å] and angles [°] for **1**.

**1** Cu			
Cu(4)-O(14)	1.962(4)	Cu(2)-N(8)	2.005(4)
Cu(4)-O(11)	1.942(4)	Cu(1)-O(5)	1.985(4)
Cu(4)-O(15)	2.489(4)	Cu(1)-O(4)	1.912(4)
Cu(4)-O(12)	1.950(4)	Cu(1)-O(1)	2.119(4)
Cu(4)-N(3)	2.013(4)	Cu(1)-O(3)	1.970(4)
Cu(4)-O(13)	2.261(4)	Cu(1)-O(2)	1.939(5)
Cu(2)-O(9)	1.939(4)	Cu(3)-O(20)	1.936(4)
Cu(2)-O(8)	1.949(4)	Cu(3)-O(19)	1.927(4)
Cu(2)-O(7)	1.944(4)	Cu(3)-O(16)	2.464(5)
Cu(2)-O(6)	2.592(4)	Cu(3)-O(17)	1.936(4)
Cu(2)-O(10)	2.217(4)	Cu(3)-O(18)	1.932(4)
O(14)-Cu(4)-O(15)	94.74(15)	O(10)-Cu(2)-O(6)	178.37(16)
O(14)-Cu(4)-N(3)	93.23(17)	N(8)-Cu(2)-O(6)	88.70(16)
O(14)-Cu(4)-O(13)	86.64(16)	N(8)-Cu(2)-O(10)	89.76(17)
O(11)-Cu(4)-O(14)	171.53(17)	O(5)-Cu(1)-O(1)	100.77(16)
O(11)-Cu(4)-O(15)	77.42(15)	O(4)-Cu(1)-O(5)	89.57(18)
O(11)-Cu(4)-O(12)	90.75(17)	O(4)-Cu(1)-O(1)	92.38(17)
O(11)-Cu(4)-N(3)	90.03(17)	O(4)-Cu(1)-O(3)	92.29(18)
O(11)-Cu(4)-O(13)	101.24(16)	O(4)-Cu(1)-O(2)	174.6(2)
O(12)-Cu(4)-O(14)	86.06(16)	O(3)-Cu(1)-O(5)	151.89(17)
O(12)-Cu(4)-O(15)	90.41(15)	O(3)-Cu(1)-O(1)	107.16(17)
O(12)-Cu(4)-N(3)	179.11(19)	O(2)-Cu(1)-O(5)	85.48(19)
O(12)-Cu(4)-O(13)	90.48(16)	O(2)-Cu(1)-O(1)	90.56(18)
N(3)-Cu(4)-O(15)	90.19(15)	O(2)-Cu(1)-O(3)	91.1(2)
N(3)-Cu(4)-O(13)	88.94(16)	O(20)-Cu(3)-O(16)	95.86(16)
O(13)-Cu(4)-O(15)	178.40(14)	O(19)-Cu(3)-O(20)	92.31(18)
O(9)-Cu(2)-O(8)	86.01(16)	O(19)-Cu(3)-O(16)	91.60(16)
O(9)-Cu(2)-O(7)	171.37(18)	O(19)-Cu(3)-O(17)	87.55(19)
O(9)-Cu(2)-O(6)	90.39(15)	O(19)-Cu(3)-O(18)	179.16(19)
O(9)-Cu(2)-O(10)	89.14(17)	O(17)-Cu(3)-O(20)	174.10(19)
O(9)-Cu(2)-N(8)	92.92(17)	O(17)-Cu(3)-O(16)	78.25(16)
O(8)-Cu(2)-O(6)	87.71(16)	O(18)-Cu(3)-O(20)	87.83(18)
O(8)-Cu(2)-O(10)	93.81(17)	O(18)-Cu(3)-O(16)	89.22(17)
O(8)-Cu(2)-N(8)	176.25(18)	O(18)-Cu(3)-O(17)	92.41(19)
O(7)-Cu(2)-O(8)	91.53(17)	N(1)-O(5)-Cu(1)	119.8(3)
O(7)-Cu(2)-O(6)	81.24(16)	N(6)-O(15)-Cu(4)	129.2(3)
O(7)-Cu(2)-O(10)	99.28(17)	N(2)-O(6)-Cu(2)	144.1(4)
O(7)-Cu(2)-N(8)	89.00(18)	N(7)-O(16)-Cu(3)	141.2(4)

Table S2. Selected bond lengths [Å] and angles [°] for **2**.

**2** GdCu			
Gd-O(4)	2.373(7)	Cu(2)-O(14)	1.953(8)
Gd-O(1)	2.406(6)	Cu(2)-O(11)	1.940(7)
Gd-O(2)	2.373(7)	Cu(2)-O(12)	1.952(8)
Gd-O(5)	2.387(6)	Cu(2)-O(13)	2.249(8)
Gd-O(6)	2.358(7)	Cu(2)-N(8)	2.034(8)
Gd-O(19)	2.364(7)	O(19)-N(6)	1.326(10)
Gd-O(15)	2.367(7)	O(15)-N(1)	1.316(11)
Gd-O(3)	2.415(7)	O(20)-N(7)	1.269(10)
Cu(1)-O(8)	1.975(7)	O(18)-N(5)	1.281(11)
Cu(1)-O(7)	1.945(7)	O(22)-N(10)	1.270(12)
Cu(1)-O(10)	2.202(7)	O(16)-N(2)	1.269(10)
Cu(1)-O(9)	1.930(6)	O(17)-N(4)	1.278(13)
Cu(1)-N(3)	2.003(8)	N(9)-O(21)	1.274(14)
O(4)-Gd-O(1)	146.9(2)	O(15)-Gd-O(2)	71.4(2)
O(4)-Gd-O(5)	77.1(2)	O(15)-Gd-O(5)	72.6(2)
O(4)-Gd-O(3)	71.8(3)	O(15)-Gd-O(3)	144.5(2)
O(1)-Gd-O(3)	132.0(3)	O(8)-Cu(1)-O(10)	95.6(3)
O(2)-Gd-O(4)	74.3(2)	O(8)-Cu(1)-N(3)	169.0(3)
O(2)-Gd-O(1)	72.6(2)	O(7)-Cu(1)-O(8)	90.6(3)
O(2)-Gd-O(5)	133.2(2)	O(7)-Cu(1)-O(10)	96.6(3)
O(2)-Gd-O(3)	128.7(2)	O(7)-Cu(1)-N(3)	88.8(3)
O(5)-Gd-O(1)	127.2(2)	O(9)-Cu(1)-O(8)	88.9(3)
O(5)-Gd-O(3)	73.5(2)	O(9)-Cu(1)-O(7)	174.1(3)
O(6)-Gd-O(4)	139.7(2)	O(9)-Cu(1)-O(10)	89.3(3)
O(6)-Gd-O(1)	73.4(2)	O(9)-Cu(1)-N(3)	90.5(3)
O(6)-Gd-O(2)	145.9(2)	N(3)-Cu(1)-O(10)	95.4(3)
O(6)-Gd-O(5)	71.9(2)	O(14)-Cu(2)-O(13)	87.2(3)
O(6)-Gd-O(19)	88.7(2)	O(14)-Cu(2)-N(8)	88.9(3)
O(6)-Gd-O(15)	103.7(2)	O(11)-Cu(2)-O(14)	172.9(3)
O(6)-Gd-O(3)	75.2(2)	O(11)-Cu(2)-O(12)	91.6(3)
O(19)-Gd-O(4)	103.6(2)	O(11)-Cu(2)-O(13)	98.8(3)
O(19)-Gd-O(1)	69.8(2)	O(11)-Cu(2)-N(8)	87.0(3)
O(19)-Gd-O(2)	77.7(2)	O(12)-Cu(2)-O(14)	91.6(3)
O(19)-Gd-O(5)	145.6(2)	O(12)-Cu(2)-O(13)	95.3(3)
O(19)-Gd-O(15)	141.0(2)	O(12)-Cu(2)-N(8)	171.4(3)
O(19)-Gd-O(3)	74.2(2)	N(8)-Cu(2)-O(13)	93.3(3)
O(15)-Gd-O(4)	90.5(2)	N(6)-O(19)-Gd	131.9(5)
O(15)-Gd-O(1)	78.6(2)	N(1)-O(15)-Gd	134.3(6)

Table S3. Selected bond lengths [Å] and angles [°] for **3**.

**3** TbCu			
Tb(1)-O(4)	2.366(5)	Cu(2)-O(14)	1.956(5)
Tb(1)-O(1)	2.380(5)	Cu(2)-O(11)	1.945(5)
Tb(1)-O(2)	2.366(5)	Cu(2)-O(12)	1.955(6)
Tb(1)-O(5)	2.389(5)	Cu(2)-O(13)	2.253(6)
Tb(1)-O(6)	2.366(5)	Cu(2)-N(8)	2.020(6)
Tb(1)-O(19)	2.355(5)	O(19)-N(6)	1.313(7)
Tb(1)-O(15)	2.369(5)	O(15)-N(1)	1.304(8)
Tb(1)-O(3)	2.398(5)	O(20)-N(7)	1.279(8)
Cu(1)-O(8)	1.978(5)	O(18)-N(5)	1.271(8)
Cu(1)-O(7)	1.945(5)	O(22)-N(10)	1.258(9)
Cu(1)-O(10)	2.201(5)	O(16)-N(2)	1.283(8)
Cu(1)-O(9)	1.944(5)	O(17)-N(4)	1.298(10)
Cu(1)-N(3)	2.012(5)	N(9)-O(21)	1.278(11)
O(4)-Tb(1)-O(1)	146.85(17)	O(15)-Tb(1)-O(1)	78.39(17)
O(4)-Tb(1)-O(5)	76.89(17)	O(15)-Tb(1)-O(5)	72.57(17)
O(4)-Tb(1)-O(15)	90.68(18)	O(15)-Tb(1)-O(3)	144.67(18)
O(4)-Tb(1)-O(3)	71.86(18)	O(8)-Cu(1)-O(10)	95.4(2)
O(1)-Tb(1)-O(5)	127.33(17)	O(8)-Cu(1)-N(3)	167.9(2)
O(1)-Tb(1)-O(3)	131.97(17)	O(7)-Cu(1)-O(8)	91.0(2)
O(2)-Tb(1)-O(4)	74.45(17)	O(7)-Cu(1)-O(10)	95.2(2)
O(2)-Tb(1)-O(1)	72.40(17)	O(7)-Cu(1)-N(3)	88.6(2)
O(2)-Tb(1)-O(5)	133.07(17)	O(9)-Cu(1)-O(8)	88.6(2)
O(2)-Tb(1)-O(15)	71.44(17)	O(9)-Cu(1)-O(7)	174.6(2)
O(2)-Tb(1)-O(3)	128.73(18)	O(9)-Cu(1)-O(10)	90.1(2)
O(5)-Tb(1)-O(3)	73.62(17)	O(9)-Cu(1)-N(3)	90.6(2)
O(6)-Tb(1)-O(4)	139.53(17)	N(3)-Cu(1)-O(10)	96.6(2)
O(6)-Tb(1)-O(1)	73.61(16)	O(14)-Cu(2)-O(13)	87.0(2)
O(6)-Tb(1)-O(2)	145.96(17)	O(14)-Cu(2)-N(8)	89.1(2)
O(6)-Tb(1)-O(5)	71.85(17)	O(11)-Cu(2)-O(14)	173.1(2)
O(6)-Tb(1)-O(15)	103.51(17)	O(11)-Cu(2)-O(12)	91.6(2)
O(6)-Tb(1)-O(3)	75.10(17)	O(11)-Cu(2)-O(13)	98.5(2)
O(19)-Tb(1)-O(4)	103.85(17)	O(11)-Cu(2)-N(8)	86.4(2)
O(19)-Tb(1)-O(1)	69.70(16)	O(12)-Cu(2)-O(14)	92.2(2)
O(19)-Tb(1)-O(2)	77.92(17)	O(12)-Cu(2)-O(13)	94.6(3)
O(19)-Tb(1)-O(5)	145.57(17)	O(12)-Cu(2)-N(8)	171.5(3)
O(19)-Tb(1)-O(6)	88.63(17)	N(8)-Cu(2)-O(13)	93.9(2)
O(19)-Tb(1)-O(15)	141.05(16)	N(6)-O(19)-Tb(1)	132.6(4)
O(19)-Tb(1)-O(3)	74.06(17)	N(1)-O(15)-Tb(1)	134.9(4)

Table S4. Selected bond lengths [Å] and angles [°] for **4**.

**4** DyCu			
Dy-O(4)	2.347(5)	Cu(2)-O(14)	1.975(6)
Dy-O(1)	2.380(5)	Cu(2)-O(11)	1.937(6)
Dy-O(2)	2.356(5)	Cu(2)-O(12)	1.945(6)
Dy-O(5)	2.392(6)	Cu(2)-O(13)	2.259(8)
Dy-O(6)	2.347(5)	Cu(2)-N(8)	2.034(7)
Dy-O(19)	2.337(5)	O(19)-N(6)	1.319(9)
Dy-O(15)	2.358(5)	O(15)-N(1)	1.290(8)
Dy-O(3)	2.392(5)	O(20)-N(7)	1.271(8)
Cu(1)-O(8)	1.986(5)	O(18)-N(5)	1.284(9)
Cu(1)-O(7)	1.945(5)	O(22)-N(10)	1.266(10)
Cu(1)-O(10)	2.206(6)	O(16)-N(2)	1.278(9)
Cu(1)-O(9)	1.942(5)	O(17)-N(4)	1.278(11)
Cu(1)-N(3)	2.019(6)	N(9)-O(21)	1.277(12)
O(4)-Dy-O(1)	147.4(2)	O(15)-Dy-O(1)	78.05(19)
O(4)-Dy-O(2)	74.3(2)	O(15)-Dy-O(5)	72.66(19)
O(4)-Dy-O(5)	76.60(19)	O(15)-Dy-O(3)	145.0(2)
O(4)-Dy-O(15)	91.0(2)	O(8)-Cu(1)-O(10)	95.8(2)
O(4)-Dy-O(3)	72.0(2)	O(8)-Cu(1)-N(3)	167.4(2)
O(1)-Dy-O(5)	127.08(19)	O(7)-Cu(1)-O(8)	91.6(2)
O(1)-Dy-O(3)	131.63(19)	O(7)-Cu(1)-O(10)	95.0(2)
O(2)-Dy-O(1)	73.12(19)	O(7)-Cu(1)-N(3)	88.1(2)
O(2)-Dy-O(5)	132.60(19)	O(9)-Cu(1)-O(8)	88.7(2)
O(2)-Dy-O(15)	71.35(19)	O(9)-Cu(1)-O(7)	174.9(2)
O(2)-Dy-O(3)	128.8(2)	O(9)-Cu(1)-O(10)	90.0(2)
O(5)-Dy-O(3)	73.67(19)	O(9)-Cu(1)-N(3)	90.6(2)
O(6)-Dy-O(4)	139.91(19)	N(3)-Cu(1)-O(10)	96.8(2)
O(6)-Dy-O(1)	72.71(19)	O(14)-Cu(2)-O(13)	86.5(3)
O(6)-Dy-O(2)	145.77(19)	O(14)-Cu(2)-N(8)	88.4(3)
O(6)-Dy-O(5)	72.44(19)	O(11)-Cu(2)-O(14)	172.6(3)
O(6)-Dy-O(15)	103.16(19)	O(11)-Cu(2)-O(12)	92.0(3)
O(6)-Dy-O(3)	75.25(19)	O(11)-Cu(2)-O(13)	98.8(3)
O(19)-Dy-O(4)	103.47(19)	O(11)-Cu(2)-N(8)	86.1(3)
O(19)-Dy-O(1)	70.21(18)	O(12)-Cu(2)-O(14)	92.5(3)
O(19)-Dy-O(2)	77.93(19)	O(12)-Cu(2)-O(13)	95.8(3)
O(19)-Dy-O(5)	145.71(19)	O(12)-Cu(2)-N(8)	170.5(3)
O(19)-Dy-O(6)	88.83(19)	N(8)-Cu(2)-O(13)	93.7(3)
O(19)-Dy-O(15)	140.92(19)	N(6)-O(19)-Dy	133.1(5)
O(19)-Dy-O(3)	73.89(19)	N(1)-O(15)-Dy	134.3(5)

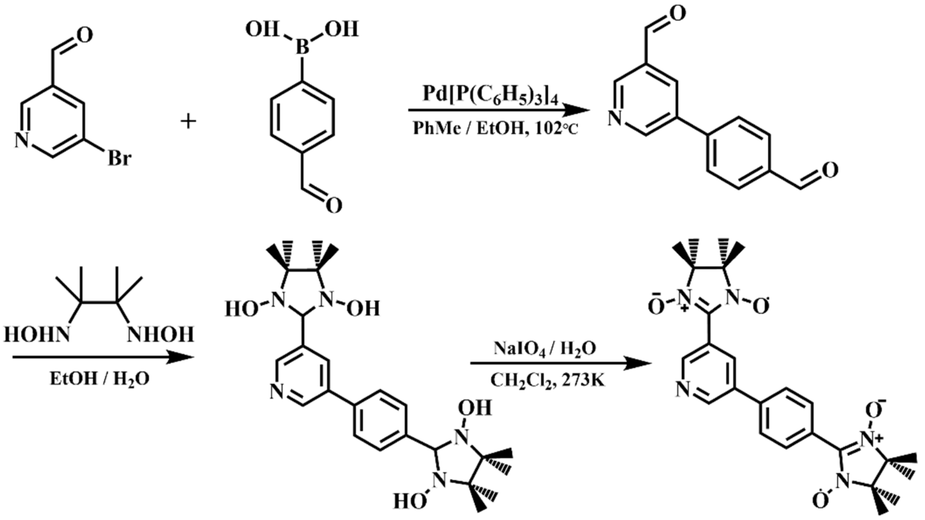

Scheme S1. The synthesis of bi-NIT-3Py-5-Ph radical ligand.

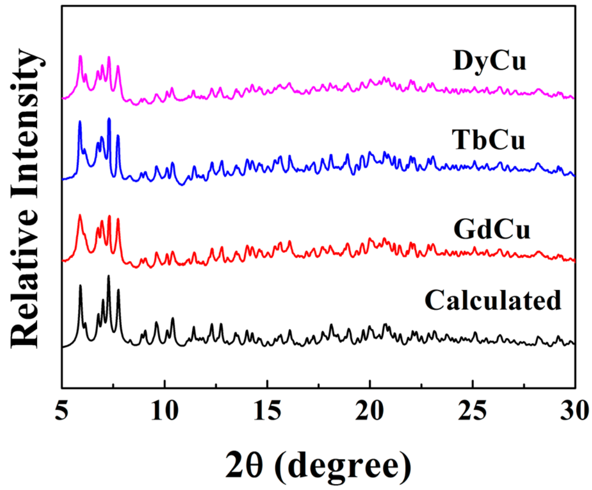

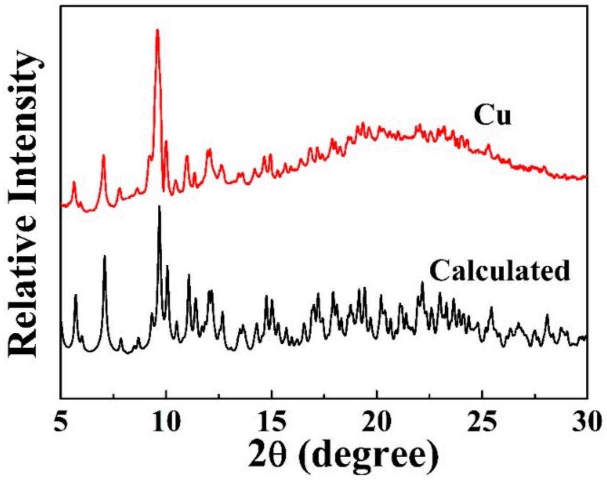

Figure S1. The Powder X-ray diffraction (PXRD) patterns for all complexes at room temperature.

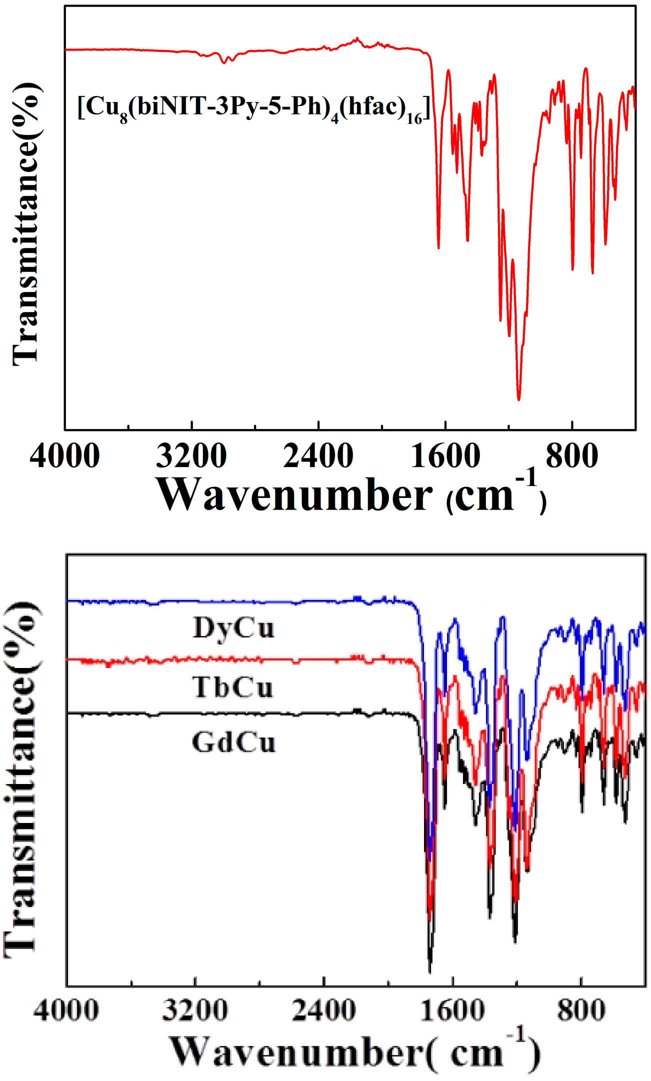

Figure S2. The IR spectra for complexes **1-4**.

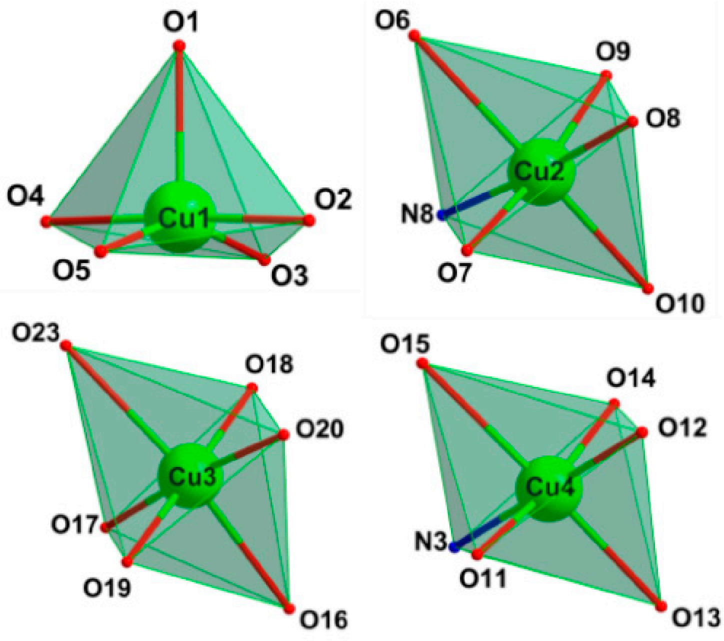

Figure S3. The coordination polyhedra of Cu^{II} ions.

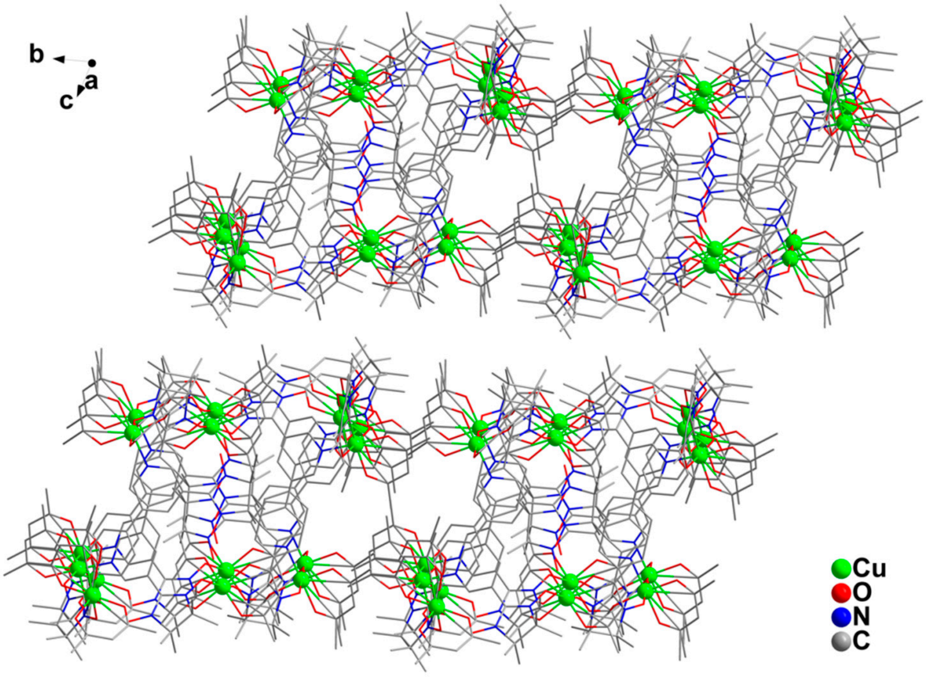

Figure S4. Packing diagram of complex **1** (Fluorine and Hydrogen atoms are omitted for the sake of clarity).

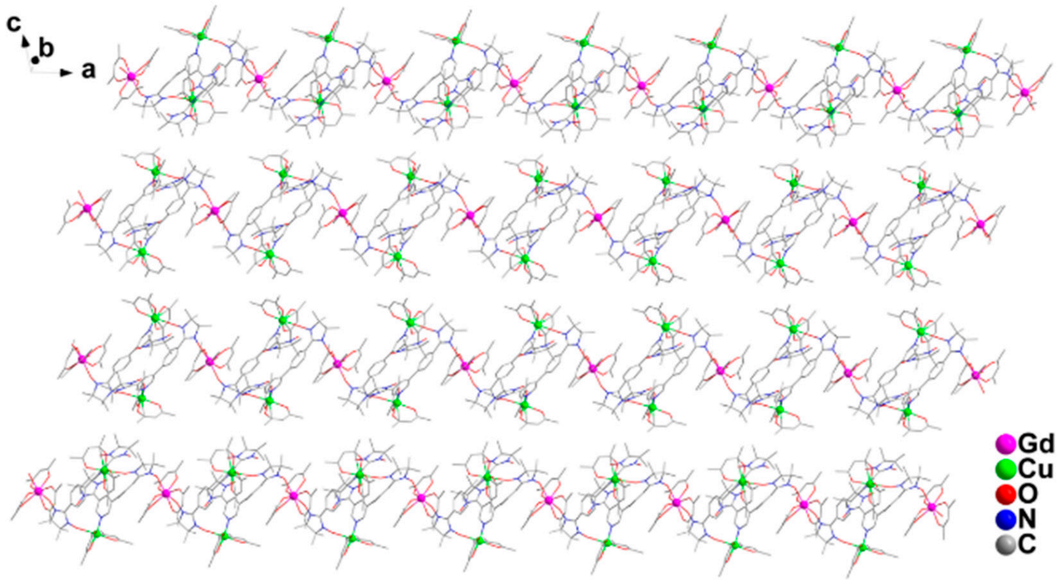

Figure S5. Packing diagram of complex **2**. (Hydrogen and fluorine atoms are not shown for the sake of clarity).

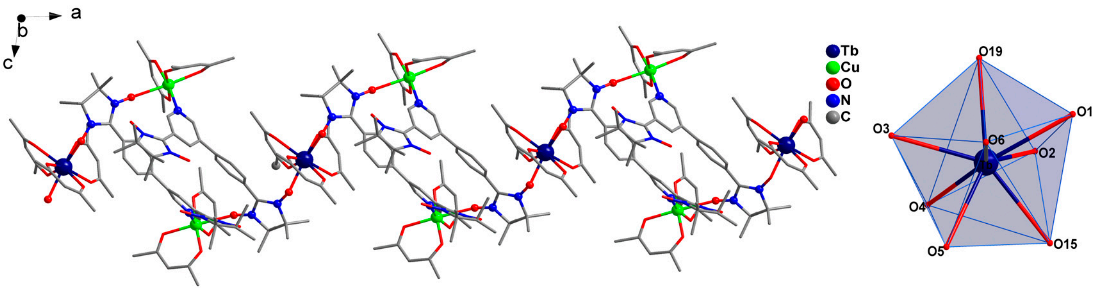

Figure S6. One-dimensional structure of **3** and local coordination geometry of Tb^{III} ion (Fluorine and Hydrogen atoms are omitted for the sake of clarity).

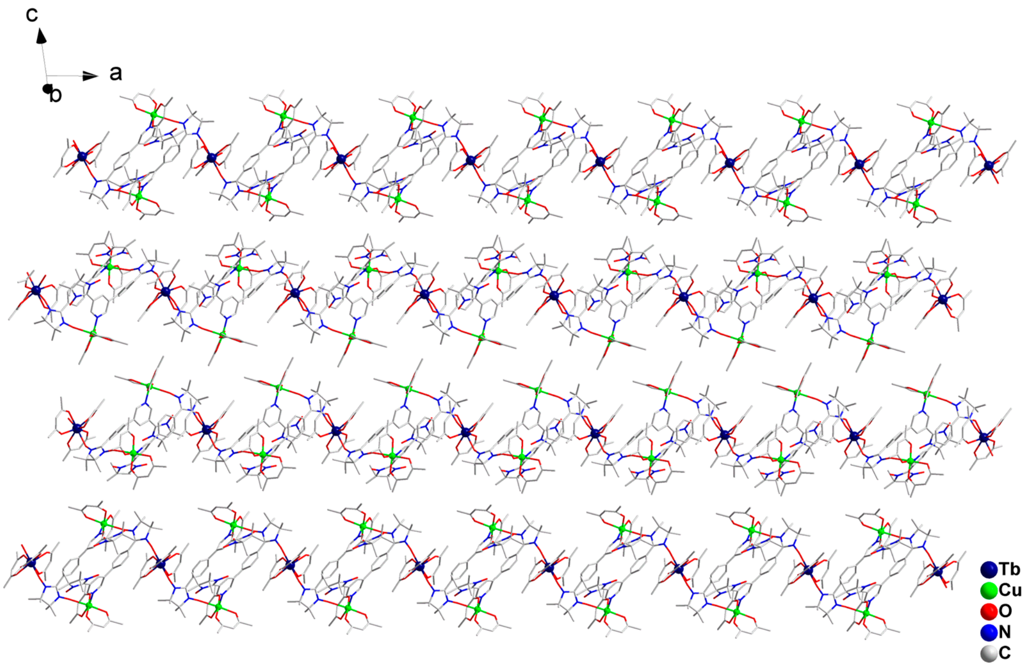

Figure S7. Packing diagram of complex **3** (Fluorine and Hydrogen atoms are omitted for the sake of clarity).

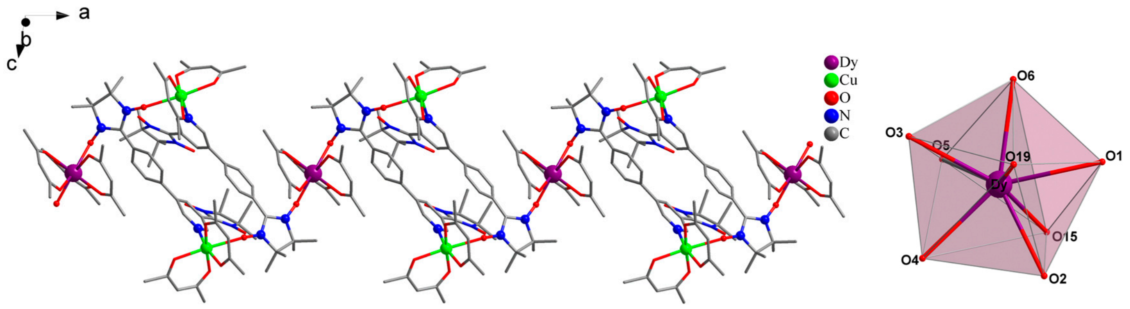

Figure S8. One-dimensional structure of **4** and local coordination geometry of Dy^{III} ion (Fluorine and Hydrogen atoms are omitted for the sake of clarity).

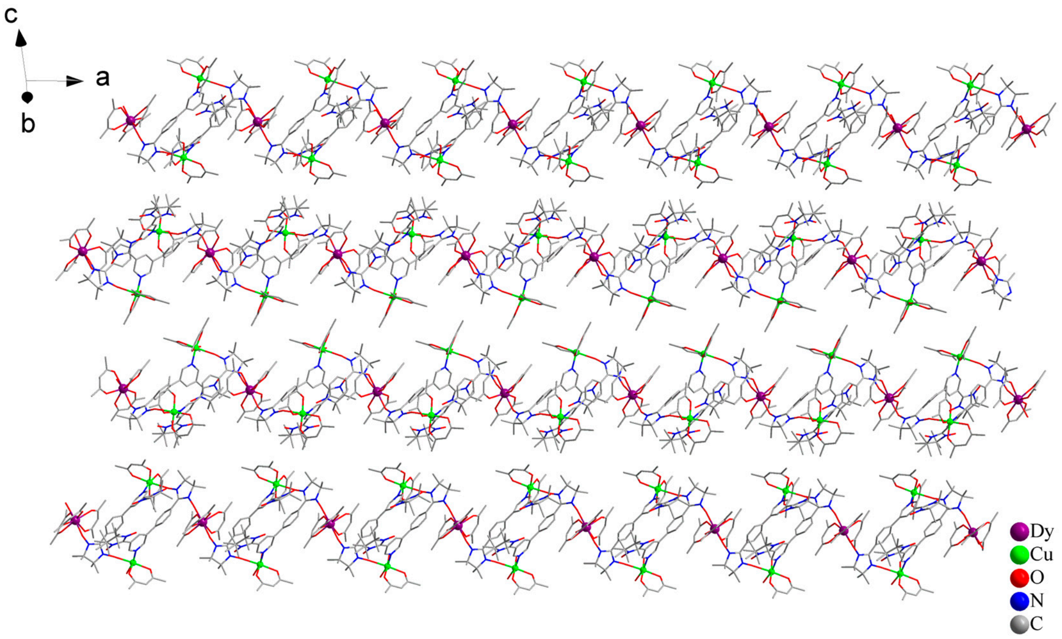

Figure S9. Packing diagram of complex **4** (Fluorine and Hydrogen atoms are omitted for the sake of clarity).

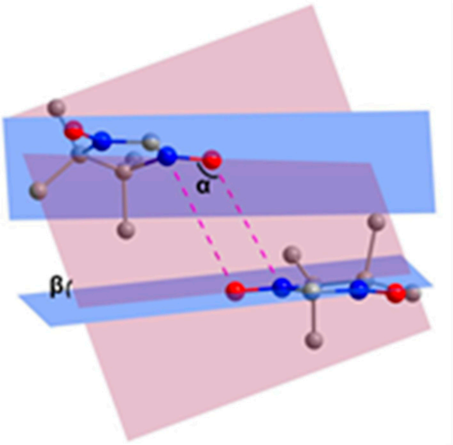

Figure S10. The relative disposition and the close contacts between the uncoordinated NO groups in **2**.

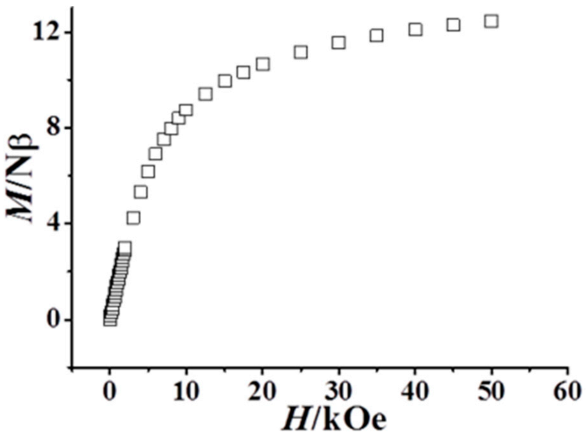

Figure S11. Field-dependent magnetization for **2** at 2.0 K.

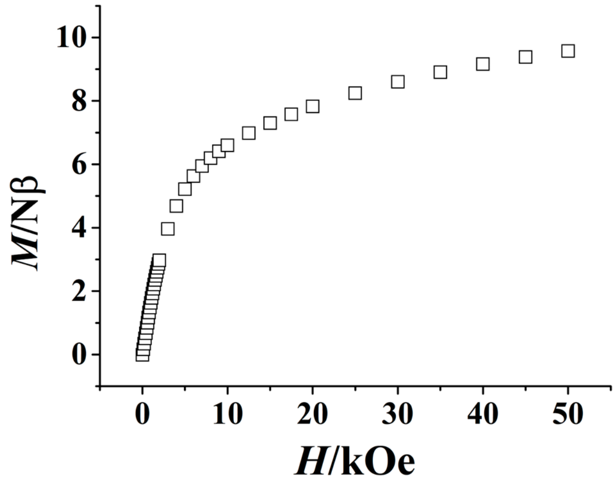

Figure S12. Field-dependent magnetization for **3** at 2.0 K.

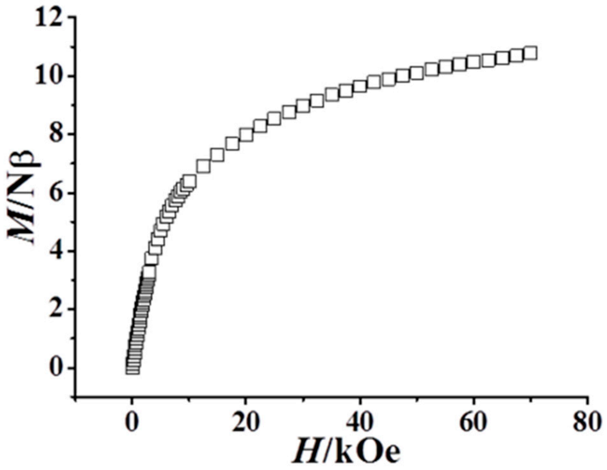

Figure S13. Field-dependent magnetization for **4** at 2.0 K.

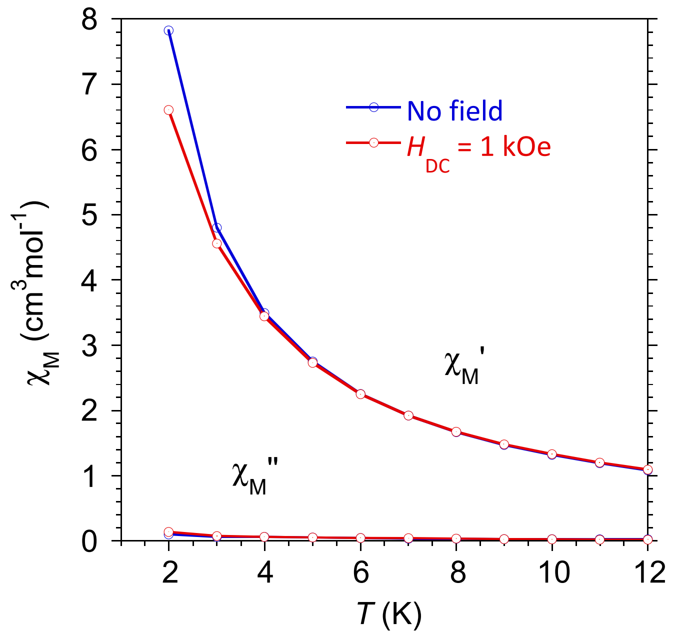

Figure S14. Frequency-dependent ac signals of the χ′ (top) and χ″ (bottom) under 1KOe dc field for compound **3**.

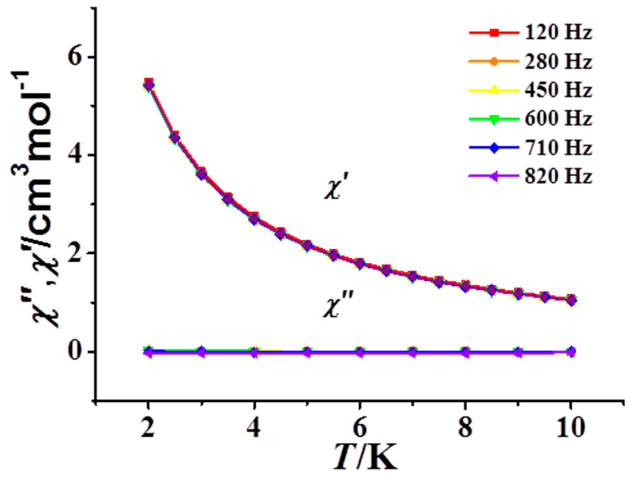

Figure S15. Frequency-dependent ac signals of the χ′ (top) and χ″ (bottom) for compound **4**.
